# Prevalence and clinical impact of hepatic steatosis on autoimmune liver disease: A systematic review and meta-analysis

**DOI:** 10.1097/HC9.0000000000000959

**Published:** 2026-04-24

**Authors:** Jarell Jie-Rae Tan, Ellina Lytvyak, Joo Wei Ethan Quek, Corrine Lee Singh, Yuan Jie Aidan Low, Shi Jie Ong, Mark Muthiah, Yu Jun Wong, Aldo J. Montano-Loza

**Affiliations:** 1 Yong Loo Lin School of Medicine, National University of Singapore, Singapore; 2 Division of Gastroenterology and Hepatology, University of Alberta, Edmonton, Alberta, Canada; 3 Division of Gastroenterology and Hepatology, Department of Medicine, National University Hospital, Singapore; 4 Department of Gastroenterology and Hepatology, Changi General Hospital, Singapore; 5 Duke–NUS Medical School, Singapore

**Keywords:** autoimmune hepatitis, hepatic steatosis, primary biliary cholangitis, primary sclerosing cholangitis

## Abstract

**Background:**

The clinical impact of hepatic steatosis (HS) among patients with autoimmune liver disease (AILD) remains unclear. We aim to determine the prevalence of HS and its clinical impact on treatment response and outcomes in patients with AILD.

**Methods:**

We systematically searched 3 electronic databases until 17 December 2025, including all studies that reported the prevalence, clinical impact, and treatment response of AILD patients with concomitant HS. The temporal trend of HS prevalence was analyzed using a quasi-Poisson regression model, with annual percent changes (APC, %) calculated.

**Results:**

Overall, 44 studies, comprising 19,898 patients with autoimmune hepatitis (AIH), primary biliary cholangitis (PBC), and primary sclerosing cholangitis (PSC) were included. The pooled prevalence of HS in patients with AIH, PBC, and PSC was 27.3%, 32.9%, and 21.6%, respectively. HS prevalence has significantly increased among PBC patients since 2010 (APC: +37.4%). While concomitant HS was associated with a higher risk of hepatic decompensation (OR: 1.6, 95% CI: 1.3–2.1, *I* ^2^=0%) and hepatocellular carcinoma (OR: 1.8, 95% CI: 1.3–2.6, *I* ^2^=0%) in patients with AIH, HS did not influence the clinical outcomes in patients with PBC. Treatment response in AIH and PBC was not influenced by concomitant HS. Available data on PSC with concomitant HS were insufficient to assess its association with clinical outcomes.

**Conclusions:**

AIH patients with concomitant HS had worse outcomes than those without HS; whereas HS did not influence the clinical outcomes in patients with PBC. Future research evaluating the impact of HS on PSC and overlap syndrome is much needed.

## Graphical abstract

**Figure:**
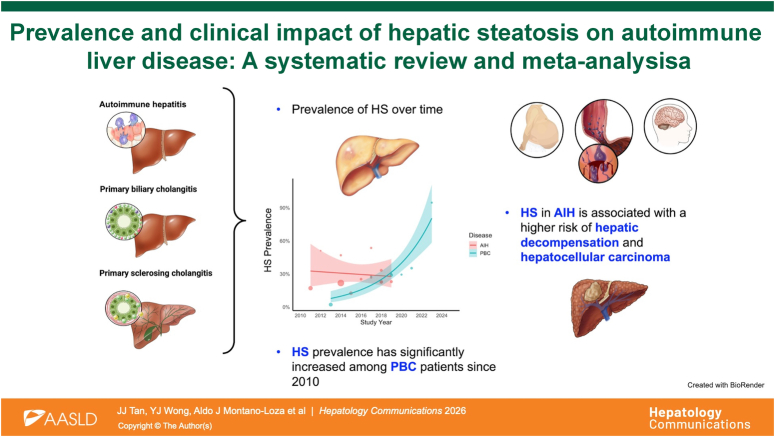

## INTRODUCTION

Autoimmune liver diseases (AILDs) are a group of acute and chronic immune-mediated progressive liver and/or biliary tract conditions that include autoimmune hepatitis (AIH), primary biliary cholangitis (PBC), and primary sclerosing cholangitis (PSC). Their global epidemiology is characterized by a relatively low prevalence that varies considerably across the world, with prevalence rates ranging from 4.2 to 29.6 cases per 100,000 person-years for AIH, from 1.9 to 57.8 for PBC, and between 0 and 16.2 for PSC.^1^^–^^3^ At the same time, despite the rarity of the AILDs, their incidence has been increasing gradually over the last few decades, with estimates currently yielding 2.9 cases per 100,000 person-years for AIH, 5.8 for PBC, and 2.6 for PSC.

Hepatic steatosis (HS) is characterized by the accumulation of excessive fat in hepatocytes, resulting in impaired function and lobular inflammation with hepatocyte injury. It most commonly occurs in the setting of metabolic disturbances or is related to excessive alcohol use. HS currently affects between 25% and 44% of the population worldwide and is becoming a leading cause of liver-related morbidity and mortality.^4^

Being chronic and progressive liver diseases, both AILDs and HS independently can lead to advanced fibrosis, cirrhosis, hepatobiliary malignancies, liver decompensation, and ultimately liver transplantation or liver-related mortality.^5^^,^^6^ Given the huge prevalence of HS, it would be expected that a substantial number of patients with AILD have concomitant HS. While their coexistence is increasingly recognized, the bidirectional nature of their relationship remains uncertain, and the evidence to date has been conflicting. For instance, Hernandez-Perez et al.^7^ reported that concomitant HS was associated with a lower treatment response rate and higher liver-related mortality in patients with PBC, whereas other cohorts failed to confirm these associations.^8^^,^^9^ Similarly, studies investigating the impact of HS on the risk of hepatocellular carcinoma (HCC) in patients with AIH have also produced conflicting results.^10^^–^^12^ In patients with chronic hepatitis B, concomitant HS has been associated with better outcomes, with a lower risk of cirrhosis-related complications and higher rates of seroclearance.^13^^,^^14^ Given the rising global burden of HS, it is clinically relevant to determine the prevalence and clinical impact in patients with AILD with concomitant HS. To address this gap, we performed a systematic review and meta-analysis to determine the prevalence and clinical outcomes of AILD in patients with and without HS.

## METHODS

### Search strategy and selection criteria

This systematic review and meta-analysis were reported according to the Preferred Reporting Items for Systematic Reviews and Meta-Analyses (PRISMA) guidelines (Supplemental Table S1, http://links.lww.com/HC9/C335).^15^ We searched EMBASE, PubMed, and Web of Science from inception to 17th December 2025. Our search strategy included keywords such as “primary biliary cholangitis”, “primary biliary cirrhosis”, “biliary cirrhosis”, “autoimmune hepatitis”, “primary sclerosing cholangitis”, “fatty liver”, and “hepatic steatosis”, with details summarized in Supplemental Table S2, http://links.lww.com/HC9/C335.

### Study selection

Five authors independently screened all titles and abstracts retrieved from the primary search, followed by full-text assessment to determine eligibility. We included both prospective and retrospective studies involving patients with AILDs as defined by established diagnostic criteria, provided that each study enrolled at least 20 patients. No restrictions were applied regarding language, sex, ethnicity, publication status, or publication year. Studies that reported data on prevalence, treatment response, or clinical outcomes in cohorts of AIH, PBC, PSC, or overlap syndrome patients with HS were included. In cases of multiple publications from the same cohort, study overlap was systematically managed according to the Cochrane Collaboration recommendation. To avoid duplication, we prioritized data from the most informative publication, rather than the largest sample size alone. Discrepancies during the screening process were resolved by consensus with senior authors.

### Data extraction

Data extraction was conducted independently by 2 authors using standardized forms (JJRT, JWEQ). Study-level characteristics, including the author(s), title, type, and the year of publication, geographical location (city or country), and study design, were collected. We also collected data on the age, sex, AILD nosological entity (AIH, PBC, PSC or overlap syndrome), prevalence of co-existing HS, diagnosis modality of HS, comorbidities (type 2 diabetes mellitus, hypertension, obesity), body mass index (BMI), and clinical outcomes (treatment response, liver-related events, HCC, liver transplantation, or death). When information was incomplete, attempts were made to contact the corresponding author for clarification.

### Risk of bias assessment

The quality of included studies was independently assessed by 2 authors using the Risk Of Bias Assessment In Non-randomized Studies of Intervention (ROBINS-I).^16^ Conflicts were resolved by discussion and, when necessary, adjudication by a third reviewer. To summarize the risk-of-bias findings, a traffic light plot was generated using the Risk-of-bias Visualization tool (robvis).

### Study definition

HS was defined according to a hierarchical diagnostic algorithm: histological confirmation was prioritized when available, followed by magnetic resonance imaging (MRI). In the absence of histology or MRI data, computer tomography (CT), ultrasound, or vibration-controlled transient elastography (VCTE) serve as diagnostic modalities of HS.^17^ The diagnosis of AIH, PBC, PSC, or overlap syndrome was based on established guidelines.^18^^–^^20^ Treatment response in AIH was defined as complete biochemical remission (normalization of serum aminotransferases, with or without normalization of IgG or histological remission)^21^ at 6 months after initiation of treatment. An insufficient response by 6 months was a failure to meet the previous definition.^21^ We defined treatment response in PBC as improvement in GLOBE score, as it has been shown to correlate with transplant-free survival in patients with PBC, based on prior studies.^22^^,^^23^ Treatment response to PSC was not addressed, as currently, there is no effective medical treatment.^24^ Treatment response to AIH/PBC overlap syndrome was based on Paris Criteria, requiring biochemical remission of both AIH and PBC components.^25^ Hepatic decompensation was defined as the occurrence of acute variceal bleeding, ascites, or hepatic encephalopathy.^26^

### Data analysis

Before proceeding from a systematic review to a meta-analysis, we qualitatively summarized the characteristics of the included studies to assess comparability. When sufficient homogeneity was observed, meta-analyses were performed to enhance the precision of our estimate. Prevalence data were stabilized using the Freeman–Tukey double arcsine transformation,^27^ and the pooled estimates were calculated with the DerSimonian–Laird random effects model, reported with 95% confidence intervals (95% CIs). Between-study heterogeneity was evaluated using the Cochran *Q* test and quantified by *I* ^2^ statistics, with a value >50% indicating significant heterogeneity.^28^

Subgroup analysis for HS prevalence was performed based on study design, publication type, study period, geographical region, method of HS diagnosis (biopsy vs. non-biopsy),^13^ risk of bias, and population size. Meta-regression was used to examine the association between metabolic risk factors and HS prevalence in AILD cohorts. To evaluate temporal trends, we extracted year-specific prevalence from included studies and adopted a quasi-Poisson regression model to account for overdispersion in count data. Models were weighted by study population size to adjust for variations in study sizes. The β coefficients were exponentiated to calculate the annual percent change (APC) in HS prevalence, as per our prior study.^2^

The “leave-one-out” method was used to explore potential bias from dominant studies. Publication bias was assessed through visual inspection of the funnel plot. All analyses were performed using R version 4.0.5 (R Foundation for Statistical Computing). A 2-tailed *p*-value <0.05 was considered statistically significant.

## RESULTS

### Overview summary

From 9981 records screened, 92 full text citations were assessed, and subsequently, we included 19,898 patients from 44 studies in our analysis (Supplemental Figure S1, http://links.lww.com/HC9/C335). The pooled cohort was predominantly female (76.5%), with 27.2% having cirrhosis at the time of diagnosis (95% CI: 16.7%–39.2%, 14 studies). Autoimmune hepatitis (AIH) was the most prevalent AILD reported (n=14,225, 23 studies), followed by primary biliary cholangitis (PBC) (n=4815, 20 studies), primary sclerosing cholangitis (PSC) (n=746, 5 studies), and then AIH-PBC overlap syndrome (n=112, 2 studies). The majority followed the established criteria for the diagnosis of AILD (35 studies), and 9 used the ICD codes to diagnose AILD. Detailed study characteristics are summarized in Supplemental Table S3, http://links.lww.com/HC9/C335. Half of the included studies had a moderate risk of bias, while the remaining had a low risk of bias (Supplemental Figure S2, http://links.lww.com/HC9/C335).

### Prevalence of HS in autoimmune liver diseases

**FIGURE 1:**
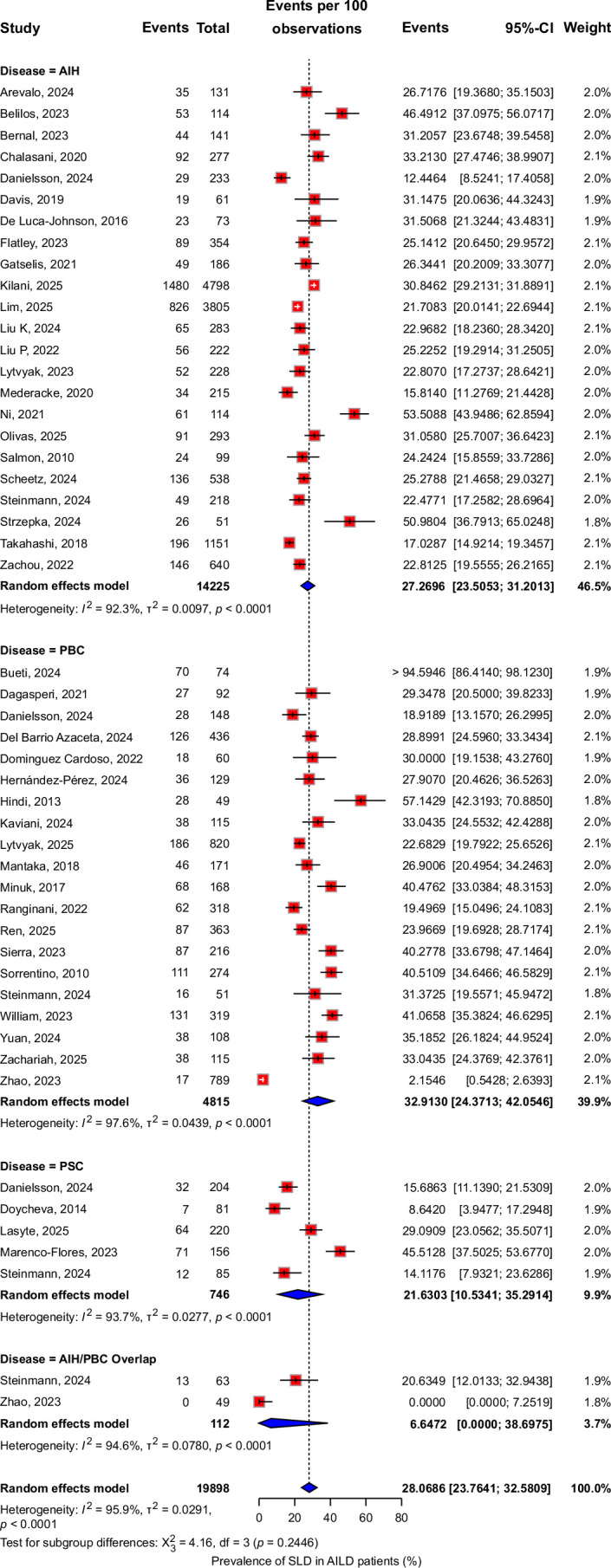
Forest plot showing the prevalence of HS in AIH, PBC, and PSC. Abbreviations: AIH, autoimmune hepatitis; HS, hepatic steatosis; PBC, primary biliary cholangitis; PSC, primary sclerosing cholangitis.

Overall, the pooled prevalence of HS in AILD was 28.1% (95% CI: 23.8%–32.6%, *I* ^2^=96%, 19,898 patients) (Figure 1), with the highest prevalence observed in PBC at 32.9% (95% CI: 24.4%–42.1%, *I* ^*2*^=98%), followed by AIH at 27.3% (95% CI: 23.5%–31.2%, *I* ^2^=92%), PSC at 21.6% (95% CI: 10.5%–35.3%, *I* ^2^=94%) and then overlap syndrome at 6.7% (95% CI: 0%–38.7%, *I* ^2^=95%). There was no significant difference in the pooled prevalence of HS across the AILD subtypes (Figure 1).

### Temporal trend

**FIGURE 2:**
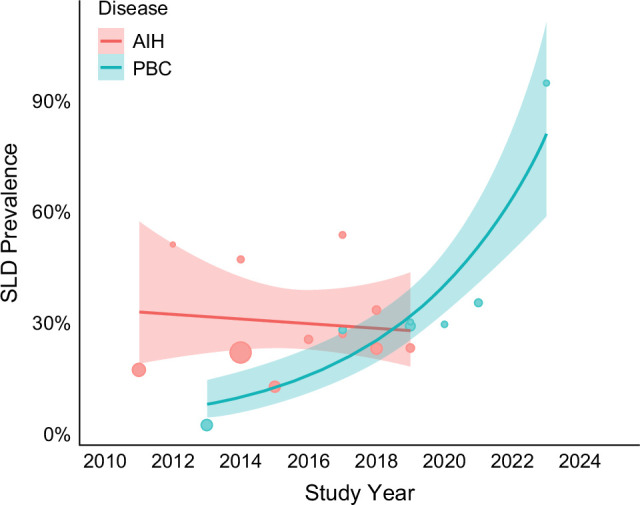
Temporal trend of HS prevalence in patients with AIH and PBC over time. Abbreviations: AIH, autoimmune hepatitis; HS, hepatic steatosis; PBC, primary biliary cholangitis; SLD, steatotic liver disease.

Since 2010, the prevalence of HS has risen disproportionately among PBC patients, with an APC of 37.4% (95% CI: 24.1%–53.8%). In contrast, the upward trend in the HS prevalence in AIH was more modest and not statistically significant (APC: 4.5%, 95% CI: −3.5% to +12.9%) (Figure 2). There is insufficient data to analyze the temporal trend for patients with PSC and overlap syndrome.

### Hepatic decompensation

**FIGURE 3:**
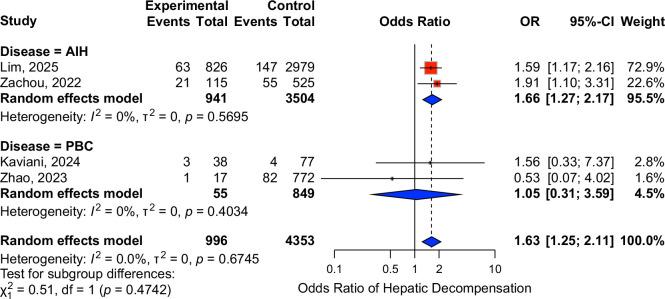
Forest plot showing the effects of HS in AIH and PBC patients on hepatic decompensation. Abbreviations: AIH, autoimmune hepatitis; CI, confidence interval; HS, hepatic steatosis; OR, odds ratio; PBC, primary biliary cholangitis.

The average follow-up duration among studies reporting outcomes of HS in AILD was 9 (range: 3–40) years. AIH patients with concomitant HS (AIH-HS) had a significantly higher pooled risk of hepatic decompensation compared with those without HS (OR: 1.66, 95% CI: 1.27–2.17, n=4445 patients), with minimal heterogeneity (*I* ^2^=0%). In contrast, concomitant HS did not influence the risk of hepatic decompensation in patients with PBC (OR: 1.05, 95% CI: 0.31–3.59, 904 patients), with minimal heterogeneity (*I* ^2^=0%) (Figure 3). No studies reported clinical outcomes of PSC and overlap syndrome patients with and without concomitant HS.

### Hepatocellular carcinoma

**FIGURE 4:**
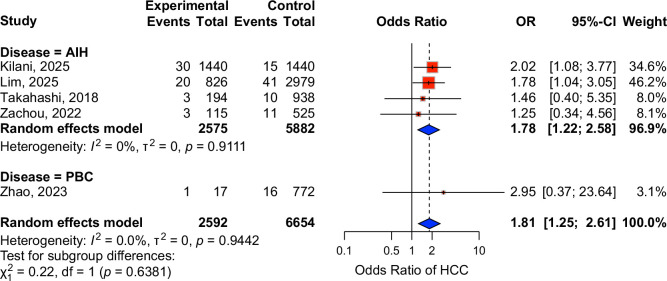
Forest plot showing the effects of HS in AIH and PBC patients on hepatocellular carcinoma. Abbreviations: AIH, autoimmune hepatitis; CI, confidence interval; HCC, hepatocellular carcinoma; HS, hepatic steatosis; OR, odds ratio; PBC, primary biliary cholangitis.

AILD patients with concomitant HS had a higher risk of hepatocellular carcinoma (HCC) (OR: 1.8, 95% CI: 1.3–2.6, 9246 patients), with minimal heterogeneity (*I* ^2^=0%) (Figure 4). This association was mainly driven by the AIH subgroup. (OR: 1.8, 95% CI: 1.2–2.6, *I* ^2^=0%, 8457 patients).

### Liver transplantation

The presence of HS did not affect the odds of undergoing liver transplantation in patients with AILD (OR: 0.70, 95% CI: 0.46–1.04, 5083 patients), with minimal heterogeneity (*I* ^2^=0%) (Supplemental Figure S3, http://links.lww.com/HC9/C335).

### All-cause mortality

Overall, co-existing HS did not influence the risk of all-cause mortality in AILD patients (OR: 0.9, 95% CI: 0.5–1.5, *I* ^2^=44%, 3968 patients) (Supplemental Figure S4, http://links.lww.com/HC9/C335).

### Treatment response

Concomitant HS did not influence the odds of achieving treatment response in patients with AIH (OR: 1.3, 95% CI: 0.4–4.3, *I* ^2^=0%, 106 patients) or PBC (OR: 0.9, 95% CI: 0.3–2.4, *I* ^2^=86%, 1165 patients) (Supplemental Figure S5, http://links.lww.com/HC9/C335).

### Sensitivity analysis

Serial exclusion of individual studies did not alter the pooled estimate of HS prevalence in AILD (Supplemental Figure S6, http://links.lww.com/HC9/C335). Subgroup analysis among patients with AIH showed a significantly higher pooled prevalence of HS among studies with a smaller population size (33.8% vs. 24.1%, *p*=0.0262) (Table 1). In patients with PBC, the pooled prevalence did not significantly differ by study design, publication type, study period, geographical region, method of HS diagnosis, risk of bias, or population size. Sensitivity analysis showed the prevalence of HS did not differ between the diagnostic methods of HS (biopsy vs. non-biopsy) (Supplemental Figure S7, http://links.lww.com/HC9/C335). Meta-regression showed the prevalence of HS was not associated with the burden of metabolic risk factors at the study level (Supplemental Figure S8, http://links.lww.com/HC9/C335).

**TABLE 1:** Prevalence of hepatic steatosis in autoimmune liver disease patients

	No. of studies	No. of AILD patients	Prevalence of HS (%)	95% CI	*I* ^2^	*p*-value for subgroup analysis
Overall	44	19,898	28.1	23.8–32.6	95.9	—
Autoimmune liver disease						
Autoimmune hepatitis (AIH)	23	14,225	27.3	23.5–31.2	92.3	0.2446
Primary biliary cholangitis (PBC)	20	4815	32.9	24.4–42.1	97.6	
Primary sclerosing cholangitis (PSC)	5	746	21.6	10.5–35.3	93.7	
Overlap syndrome (AIH-PBC OS)	2	112	6.7	0–38.7	94.6	
Autoimmune hepatitis (AIH)						
Study design						
Retrospective	21	14,023	27.0	22.9–31.2	92.9	0.2689
Prospective	2	202	31.2	24.9–37.8	0	
Publication type						
Abstract	9	2131	26.6	21.2–32.6	85.8	0.7746
Full text	14	12,094	27.8	22.6–33.3	94.3	
Study period						
Before 2020	6	1876	24.5	18.1–31.5	88.9	0.3836
After 2020	17	12,349	28.2	23.6–33.0	92.2	
Geographical region^a^						
North America	9	6281	32.5	27.2–38.0	78.3	0.0557
South America	1	131	26.7	19.4–35.2	—	
Europe	7	1598	22.2	17.4–27.4	83.5	
Asia	5	5575	27.0	16.3–39.3	94.2	
Method of HS diagnosis^b^						
Biopsy	13	3616	24.7	20.2–29.5	86.5	0.5978
Non-biopsy	6	9868	26.4	22.8–30.2	95.0	
Risk of bias						
Low	13	11,989	25.9	20.1–32.2	95.1	0.3764
Moderate	10	2236	29.2	25.4–33.2	70.2	
Population size						
<200	9	1935	33.8	25.6–42.6	93.8	**0.0262**
>200	14	12,990	24.1	21.2–27.1	91.5	
Primary biliary cholangitis (PBC)						
Study design						
Retrospective	19	4599	32.5	23.6–42.1	97.7	0.1905
Prospective	1	216	40.3	33.8–46.9	—	
Publication type						
Abstract	10	2576	36.2	22.3–51.4	96.3	0.4772
Full text	10	2239	29.6	19.7–40.6	98.1	
Study period						
Before 2020	4	662	40.1	29.2–51.6	83.2	0.2522
After 2020	16	4153	31.1	21.1–42.0	97.9	
Geographical region^a^						
North America	7	1245	36.5	28.2–45.1	89.8	0.3455
Europe	9	1490	37.2	22.0–53.8	95.6	
Asia	3	1260	17.3	1.8–43.2	98.9	
Method of HS diagnosis^b^						
Biopsy	7	990	33.8	25.4–42.9	85.7	0.7915
Non-biopsy	10	3386	31.3	16.0–49.0	98.6	
Risk of bias						
Low	9	2124	28.7	17.7–41.2	98.3	0.3998
Moderate	11	2691	36.4	23.9–49.8	95.7	
Population size						
<200	9	913	41.3	25.8–57.7	95.0	0.1099
>200	11	3902	26.7	18.4–35.8	98.1	

Bold values are statistically significant subgroup differences.

^a^ Studies with mixed (biopsy + non-biopsy) methods of HS diagnosis were excluded from the subgroup analysis.

^b^ Studies spanning >1 continent were excluded from subgroup analysis.

Abbreviations: AILD, autoimmune liver disease; CI, confidence interval; HS, hepatic steatosis.

### Publication bias

Visual inspection of the funnel plot on the prevalence of HS in AIH did not suggest significant asymmetry, and the Egger test confirmed no significant small-study effect (*p*=0.661) (Supplemental Figure S9, http://links.lww.com/HC9/C335). Funnel plot on the pooled prevalence of HS in PBC suggests publication bias based on 2 outlier studies,^29^^,^^30^ and a significant Egger test (*p*=0.0061, Supplemental Figure S10, http://links.lww.com/HC9/C335).

## DISCUSSION

In this systematic review and meta-analysis, we examined the prevalence and clinical impact of concomitant hepatic steatosis (HS) in autoimmune liver diseases (AILDs). Our study provides several novel insights: First, we found that the overall pooled prevalence of HS among AILD (28%) mirrors that of the general population, and its prevalence did not differ significantly across AILD subtypes.^31^ Second, concomitant HS was associated with a substantially higher risk of hepatic decompensation and HCC in patients with AIH. Finally, concomitant HS did not significantly affect treatment response in AIH and PBC.

Given the rising global prevalence of steatotic liver disease (25%–44%), a HS prevalence of ~27% in AIH likely reflects the broader metabolic landscape rather than a disease-specific increase. Several factors may explain why HS prevalence in AIH does not exceed that of the general population. First, in many included studies, HS was assessed at or near the time of AIH diagnosis. At this stage, patients may not yet have experienced sufficient corticosteroid exposure to develop clinically detectable steatosis; consequently, steroid-induced HS may be underrepresented in cross-sectional baseline estimates. Second, treatment strategies for AIH vary substantially across studies, including differences in steroid dose, duration, and tapering protocols, as well as the use of steroid-sparing agents. Such heterogeneity may attenuate the measurable cumulative impact of corticosteroids on pooled HS prevalence. Third, patients presenting with more aggressive AIH phenotypes may have significant systemic inflammation and reduced nutritional intake before diagnosis, which could partially offset metabolic risk and limit steatosis development.

Notably, while the prevalence of HS in AIH remained stable over time, the prevalence of HS in PBC increased in more recent cohorts. This divergence may reflect a combination of factors, including the increased awareness and earlier diagnosis of PBC through highly specific autoantibody testing, improved survival with ursodeoxycholic acid, and longer life expectancy.^2^^,^^32^^,^^33^ The global rise in metabolic dysfunction–associated steatotic liver disease (MASLD), driven by increasing obesity and type 2 diabetes, likely contributes to the growing coexistence of HS in PBC, a disease that predominantly affects middle-aged and older women, where metabolic risk factors are increasingly prevalent. Enhanced detection of steatosis through widespread use of controlled attenuation parameter and cross-sectional imaging in contemporary studies may further inflate recent prevalence estimates compared with earlier biopsy-based or ultrasound-based cohorts. In contrast, AIH is frequently diagnosed earlier and managed with immunosuppression, which may attenuate inflammation-driven metabolic perturbations, potentially stabilizing HS prevalence across eras.

While concomitant dyslipidemia, contributed by cholestasis and impaired bile acid secretion, is generally considered benign with respect to cardiovascular outcomes,^29^^,^^34^ its potential effect on liver-related outcomes remains largely unknown. In our analysis, concomitant HS did not appear to adversely influence the treatment response and liver-related outcomes in patients with PBC.

One of the key findings from this study was that patients with AIH-HS demonstrated a significantly higher risk of both hepatic decompensation and HCC. The mechanism behind this association is likely multifactorial and bidirectional. Patients with difficult-to-control AIH alone may experience an inherently higher risk of hepatic decompensation and HCC. At the same time, prolonged corticosteroid therapy, often required in such cases, can also predispose to HS. Given the comparable efficacy of a lower dose of prednisolone to induce remission in AIH, a lower loading dose of steroids, particularly among rapid responders, seems reasonable to reduce unnecessary steroid exposure.^35^ To further reduce systemic exposure to corticosteroids, budesonide and faster conversion to steroid-sparing agents should be considered, especially in non-cirrhotic AIH patients.

Although AIH patients with concomitant HS had higher risks of hepatic decompensation and HCC, this did not translate into increased transplantation or all-cause mortality.^11^ This is because decompensation and HCC are intermediate events, whereas transplantation and death are later outcomes shaped by competing risks and transplant selection. A limited number of studies reporting on LT and mortality may have reduced statistical power to determine the impact of HS on these outcomes. Finally, all-cause mortality may dilute liver-specific risk due to competing non-hepatic causes of death. Closer monitoring in AIH patients may enable timely intervention, allowing earlier recognition of complications in higher-risk patients, potentially mitigating fibrosis progression and decompensation. Prospective longitudinal studies are required to determine whether metabolic optimization and risk-adapted surveillance improve long-term outcomes. Collectively, these findings suggest that HS is a clinically relevant comorbidity in AIH, with implications for both prognosis and surveillance strategies.

To our best knowledge, this is the most comprehensive meta-analysis to date evaluating the clinical burden and impact of HS across the entire spectrum of AILD. Given the rising global prevalence of HS, our findings are highly relevant: HS is common in AILD, and importantly, its presence is associated with an increased risk of hepatic decompensation and HCC, particularly in AIH. Nonetheless, the interpretation of our findings should be interpreted within the limitations of the study. Substantial heterogeneity in the pooled prevalence of HS likely reflects variation in diagnostic approaches across included studies and therefore raises the possibility of misclassification bias. However, sensitivity analysis showed no significant difference in pooled prevalence between both biopsy and non-biopsy diagnoses of HS (Supplemental Figure S6, http://links.lww.com/HC9/C335), supporting the overall robustness of the estimate. The small number of studies reporting treatment outcomes limits power for subgroup analyses and constrains inference about therapeutic effects; nevertheless, these pooled data establish a reference point for treatment response rates that can inform comparative assessments as the evidence base grows. Definitions of treatment response were heterogeneous, which reduces cross-study comparability and should be acknowledged when interpreting pooled estimates. Most studies predate the recent nomenclature change of steatotic liver disease, and only 2 studies^29^^,^^36^ quantified alcohol exposure, limiting our ability to decipher HS subtypes. Similarly, data is sparse for PSC and overlap syndrome with concomitant HS, and longitudinal assessments that capture both baseline and incident steatosis during treatment are uncommon, restricting evaluation of long-term complication risk in autoimmune liver disease. Given that most studies reported unadjusted, raw events rather than adjusted hazard ratios to account for confounding, we cannot exclude residual confounding in study outcomes from a true causal relationship. Despite these caveats, the systematic approach and sensitivity analyses strengthen confidence in the pooled prevalence estimates. Our study highlighted specific gaps such as standardized diagnostic criteria, alcohol quantification, harmonized outcome definitions, and longitudinal outcome data. Future individual patient-data meta-analyses with standardized fibrosis assessment are required to determine whether HS independently predicts adverse outcomes in AIH after adjustment for baseline disease severity.^37^^–^^39^

In conclusion, our study revealed that approximately one-third of patients with AILD have concomitant HS, with its prevalence in PBC rising significantly over the past decade. The clinical impact of HS differs by AILD subtypes: in AIH, HS is associated with worse outcomes, including higher risks of hepatic decompensation and HCC, whereas in PBC, HS does not appear to influence the risk of hepatic decompensation. As the prevalence of both HS and AILD continues to rise globally, further studies are needed to elucidate underlying mechanisms, refine risk stratification, and guide optimal management strategies, particularly in patients with AIH-HS concomitance.

## Supplementary Material
