# Supplementary material for: Prevalence and clinical impact of hepatic steatosis on autoimmune liver disease: A systematic review and meta-analysis
Source: Hepatol Commun. 2026 Apr 24;10(5):e0959. doi: 10.1097/HC9.0000000000000959 (PMC13120599; doi:10.1097/HC9.0000000000000959)

**Prevalence and clinical impact of hepatic steatosis in patients with Autoimmune Liver Disease: A Systematic Review and Meta-analysis**

*Jarell Jie-Rae Tan, *Ellina Lytvyak, *Joo Wei Ethan Quek, Corrine Lee Singh, Yuan Jie Aidan Low, Shi Jie Ong, Mark Muthiah, ^#^Yu Jun Wong, ^#^Aldo Montano-Loza.

**SUPPLEMENTARY APPENDIX**

| *Table S1* | Preferred Reporting Items for Systematic Reviews and Meta-analysis (PRISMA) guidelines | Page 2 |
| --- | --- | --- |
| *Table S2* | Literature search strategy | Page 5 |
| *Table S3* | Baseline Characteristics of Included Studies | Page 6 |
| *Figure S1* | PRISMA flowchart | Page 9 |
| *Figure S2* | Traffic light plot summarizing risk-of-bias of all included studies | Page 10 |
| *Figure S3* | Forest plot showing the effects of hepatic steatosis in AIH and PBC patients on undergoing **liver transplantation**. | Page 11 |
| *Figure S4* | Forest plot showing the effects of hepatic steatosis in AIH and PBC patients on **all-cause mortality**. | Page 12 |
| *Figure S5* | Forest plot showing the effects of hepatic steatosis in AIH and PBC patients on **treatment response**. | Page 13 |
| *Figure S6* | Leave-one-out analysis showing serial exclusion of individual studies did not alter the pooled estimates of hepatic steatosis in AILD | Page 14 |
| *Figure S7* | Sensitivity analysis of hepatic steatosis prevalence by method of hepatic steatosis diagnosis (biopsy versus non-biopsy) in patients with A) Autoimmune Hepatitis, and B) Primary Biliary Cholangitis | Page 15 |
| *Figure S8* | Meta-regression of prevalence of hepatic steatosis in AIH and PBC against **A**: BMI (kg/m^2^); **B**: Type 2 Diabetes Mellitus; **C**: Hypertension; **D**: Dyslipidemia | Page 16 |
| *Figure S9* | Funnel Plot on the pooled prevalence of autoimmune hepatitis. | Page 17 |
| *Figure S10* | Funnel Plot on the pooled prevalence of primary biliary cholangitis. | Page 18 |

**Table S1:** Preferred Reporting Items for Systematic Reviews and Meta-analysis (PRISMA) guidelines

| **Section and Topic** | **Item #** | **Checklist item** | **Location where item is reported** |
| --- | --- | --- | --- |
| **TITLE** | | |  |
| Title | 1 | Identify the report as a systematic review. | 1 |
| **ABSTRACT** | | |  |
| Abstract | 2 | See the PRISMA 2020 for Abstracts checklist. | 3 |
| **INTRODUCTION** | | |  |
| Rationale | 3 | Describe the rationale for the review in the context of existing knowledge. | 4, 5 |
| Objectives | 4 | Provide an explicit statement of the objective(s) or question(s) the review addresses. | 4, 5 |
| **METHODS** | | |  |
| Eligibility criteria | 5 | Specify the inclusion and exclusion criteria for the review and how studies were grouped for the syntheses. | 5 |
| Information sources | 6 | Specify all databases, registers, websites, organisations, reference lists and other sources searched or consulted to identify studies. Specify the date when each source was last searched or consulted. | 5 |
| Search strategy | 7 | Present the full search strategies for all databases, registers and websites, including any filters and limits used. | 5 |
| Selection process | 8 | Specify the methods used to decide whether a study met the inclusion criteria of the review, including how many reviewers screened each record and each report retrieved, whether they worked independently, and if applicable, details of automation tools used in the process. | 5 |
| Data collection process | 9 | Specify the methods used to collect data from reports, including how many reviewers collected data from each report, whether they worked independently, any processes for obtaining or confirming data from study investigators, and if applicable, details of automation tools used in the process. | 6 |
| Data items | 10a | List and define all outcomes for which data were sought. Specify whether all results that were compatible with each outcome domain in each study were sought (e.g. for all measures, time points, analyses), and if not, the methods used to decide which results to collect. | 6 |
|  | 10b | List and define all other variables for which data were sought (e.g. participant and intervention characteristics, funding sources). Describe any assumptions made about any missing or unclear information. | NA |
| Study risk of bias assessment | 11 | Specify the methods used to assess risk of bias in the included studies, including details of the tool(s) used, how many reviewers assessed each study and whether they worked independently, and if applicable, details of automation tools used in the process. | 6 |
| Effect measures | 12 | Specify for each outcome the effect measure(s) (e.g. risk ratio, mean difference) used in the synthesis or presentation of results. | 6, 7 |
| Synthesis methods | 13a | Describe the processes used to decide which studies were eligible for each synthesis (e.g. tabulating the study intervention characteristics and comparing against the planned groups for each synthesis (item #5)). | 6, 7 |
|  | 13b | Describe any methods required to prepare the data for presentation or synthesis, such as handling of missing summary statistics, or data conversions. | 6, 7 |
|  | 13c | Describe any methods used to tabulate or visually display results of individual studies and syntheses. | 6, 7 |
|  | 13d | Describe any methods used to synthesize results and provide a rationale for the choice(s). If meta-analysis was performed, describe the model(s), method(s) to identify the presence and extent of statistical heterogeneity, and software package(s) used. | 6, 7 |
|  | 13e | Describe any methods used to explore possible causes of heterogeneity among study results (e.g. subgroup analysis, meta-regression). | 7 |
|  | 13f | Describe any sensitivity analyses conducted to assess robustness of the synthesized results. | 7 |
| Reporting bias assessment | 14 | Describe any methods used to assess risk of bias due to missing results in a synthesis (arising from reporting biases). | 7 |
| Certainty assessment | 15 | Describe any methods used to assess certainty (or confidence) in the body of evidence for an outcome. | 7 |
| **RESULTS** | | |  |
| Study selection | 16a | Describe the results of the search and selection process, from the number of records identified in the search to the number of studies included in the review, ideally using a flow diagram. | 10, Figure 1 |
|  | 16b | Cite studies that might appear to meet the inclusion criteria, but which were excluded, and explain why they were excluded. | NA |
| Study characteristics | 17 | Cite each included study and present its characteristics. | Table 1 |
| Risk of bias in studies | 18 | Present assessments of risk of bias for each included study. | Figure S1 |
| Results of individual studies | 19 | For all outcomes, present, for each study: (a) summary statistics for each group (where appropriate) and (b) an effect estimate and its precision (e.g. confidence/credible interval), ideally using structured tables or plots. | Table 2 |
| Results of syntheses | 20a | For each synthesis, briefly summarise the characteristics and risk of bias among contributing studies. | 7, 8 |
|  | 20b | Present results of all statistical syntheses conducted. If meta-analysis was done, present for each the summary estimate and its precision (e.g. confidence/credible interval) and measures of statistical heterogeneity. If comparing groups, describe the direction of the effect. | 7-9, Table 2 |
|  | 20c | Present results of all investigations of possible causes of heterogeneity among study results. | 7-9, Table 2 |
|  | 20d | Present results of all sensitivity analyses conducted to assess the robustness of the synthesized results. | 7-10, Table 2 |
| Reporting biases | 21 | Present assessments of risk of bias due to missing results (arising from reporting biases) for each synthesis assessed. | NA |
| Certainty of evidence | 22 | Present assessments of certainty (or confidence) in the body of evidence for each outcome assessed. | 8-10 |
| **DISCUSSION** | | |  |
| Discussion | 23a | Provide a general interpretation of the results in the context of other evidence. | 10-12 |
|  | 23b | Discuss any limitations of the evidence included in the review. | 11 |
|  | 23c | Discuss any limitations of the review processes used. | 11 |
|  | 23d | Discuss implications of the results for practice, policy, and future research. | 10-12 |
| **OTHER INFORMATION** | | |  |
| Registration and protocol | 24a | Provide registration information for the review, including register name and registration number, or state that the review was not registered. | The review was not registered |
|  | 24b | Indicate where the review protocol can be accessed, or state that a protocol was not prepared. | 2 |
|  | 24c | Describe and explain any amendments to information provided at registration or in the protocol. | NA |
| Support | 25 | Describe sources of financial or non-financial support for the review, and the role of the funders or sponsors in the review. | NA |
| Competing interests | 26 | Declare any competing interests of review authors. | 2 |
| Availability of data, code and other materials | 27 | Report which of the following are publicly available and where they can be found: template data collection forms; data extracted from included studies; data used for all analyses; analytic code; any other materials used in the review. | 2 |

*From:*  Page MJ, McKenzie JE, Bossuyt PM, Boutron I, Hoffmann TC, Mulrow CD, et al. PRISMA 2020 statement: an updated guideline for reporting systematic reviews. BMJ 2021;372:n71. doi: 10.1136/bmj.n71

**Table S2:** Literature search strategy

**Embase**

| #1 | 'primary biliary cirrhosis' OR 'autoimmune hepatitis' OR 'primary sclerosing cholangitis' OR "primary biliary cirrhosis" OR "primary biliary cholangitis" OR "biliary cirrhosis" OR "autoimmune hepatitis" OR "primary sclerosing cholangitis" (ti, ab, kw) |
| --- | --- |
| #2 | ‘biliary cirrhosis, primary’ OR ‘biliary cirrhosis’ OR ‘autoimmune hepatitis’ OR ‘sclerosing cholangitis, primary’ OR ‘sclerosing cholangitis’ (emtree) |
| #3 | (((fatty liver(ti, ab, kw)) OR (steatohepatitis(ti, ab, kw))) OR (steatotic liver disease(ti, ab, kw))) OR (NAFLD(ti, ab, kw))) OR (non-alcoholic steatohepatitis(ti, ab, kw))) OR (non-alcoholic fatty liver disease(ti, ab, kw)])) OR (metabolic dysfunction associated steatohepatitis(ti, ab, kw))) OR (MASLD(ti, ab, kw))) OR (MetALD(ti, ab, kw))) OR (alcoholic liver disease(ti, ab, kw)) |
| #4 | ‘Fatty liver’ OR ‘steatohepatitis’ OR ‘non-alcoholic fatty liver’ OR ‘alcohol liver disease’ OR ‘alcohol liver cirrhosis’ (emtree) |
| #5 | #1 OR #2 |
| #6 | #3 OR #4 |
| #7 | #5 AND #6 |

**PubMed**

| #1 | "primary biliary cirrhosis"[Title/Abstract] OR "biliary cirrhosis"[Title/Abstract] OR "primary biliary cholangitis"[Title/Abstract] OR "autoimmune hepatitis"[Title/Abstract] OR "primary sclerosing cholangitis"[Title/Abstract] |
| --- | --- |
| #2 | ((((((biliary cirrhosis, primary[MeSH Terms]) OR (primary biliary cirrhosis[MeSH Terms])) OR (biliary cirrhosis[MeSH Terms])) OR (autoimmune hepatitis[MeSH Terms])) OR (autoimmune hepatitides[MeSH Terms])) OR (cholangiitis, sclerosing[MeSH Terms])) OR (sclerosing cholangitis[MeSH Terms]) |
| #3 | ((((liver steatosis[MeSH Terms]) OR (liver steatosis[MeSH Terms])) OR (steatohepatitides[MeSH Terms])) OR (liver steatoses[MeSH Terms])) OR (fatty liver[MeSH Terms]) |
| #4 | (((((((((fatty liver[Title/Abstract]) OR (steatohepatitis[Title/Abstract])) OR (steatotic liver disease[Title/Abstract])) OR (NAFLD[Title/Abstract])) OR (non-alcoholic steatohepatitis[Title/Abstract])) OR (non-alcoholic fatty liver disease[Title/Abstract])) OR (metabolic dysfunction associated steatohepatitis[Title/Abstract])) OR (MASLD[Title/Abstract])) OR (MetALD[Title/Abstract])) OR (alcoholic liver disease[Title/Abstract]) |
| #5 | #1 OR #2 |
| #6 | #3 OR #4 |
| #7 | #5 AND #6 |

**Web of Science**

“Primary biliary cirrhosis” OR “primary biliary cholangitis” OR “biliary cirrhosis” OR “autoimmune hepatitis” OR “primary sclerosing cholangitis” OR “sclerosing cholangitis”

AND

“Fatty liver” OR “steatohepatitis” OR “steatotic liver disease” OR “hepatic steatosis” OR “non-alcoholic fatty liver disease” OR “non-alcoholic steatohepatitis” OR “NAFLD” OR ‘metabolic dysfunction associated steatotic liver disease’ OR ‘metabolic dysfunction associated steatohepatitis” OR “MASLD” or “metALD” OR ‘alcoholic liver disease” 

**Table S3**: Baseline characteristics of included studies

| **No** | **Author, Year** | **Country** | **Study Design** | **Study Period** | **AILD subtype** | **AILD diagnosis** | **Sample Size (n)** | **HS diagnosis method** | **HS (%)** | **Female sex (%)** | **Baseline cirrhosis (%)** | **BMI (kg/m2)*** | **Type 2 Diabetes (%)** | **Hypertension (%)** | **Dyslipidemia (%)** | **Treatment Response (%)** | **HCC (%)** | **Hepatic Decompensation (%)** |
| --- | --- | --- | --- | --- | --- | --- | --- | --- | --- | --- | --- | --- | --- | --- | --- | --- | --- | --- |
| 1 | Arevalo, 2024 | Chile | Retrospective | 2014 -2019 | AIH | Biopsy | 131 | Biopsy | 35 (26.7%) | 100 (76.0%) | NR | NR | 24 (18.3) | 35 (26.7) | NR | NR | NR | NR |
| 2 | Belilos, 2023 | USA | Retrospective | 2009 - 2018 | AIH | Biopsy | 114 | Biopsy | 53 (46.5%) | 93 (81.6%) | NR | NR | NR | NR | NR | NR | NR | NR |
| 3 | Bernal, 2023 | USA | Prospective | NR | AIH | Biopsy | 141 | Biopsy | 44 (24.8%) | 167 (90.3%) | NR | NR | 36 (19.5) | 75 (40.5) | 58 (31.4) | NR | NR | NR |
| 4 | Bueti, 2024 | Italy | Retrospective | 2023 | PBC | Clinical | 74 | VCTE | 70 (94.6%) | 68 (91.9%) | 14 (18.9%) | 25.7 (4.2) | NR | NR | NR | NR | NR | NR |
| 5 | Chalasani, 2020 | USA | Retrospective | 2017 - 2019 | AIH | Clinical | 277 | VCTE | 92 (33.2%) | 227 (82.0%) | NR | 29.7 (7.2) | NR | NR | NR | NR | NR | NR |
| 6 | Dagasperi, 2021 | Italy | Retrospective | 2020 – 2021 | PBC | Clinical | 92 | VCTE | 27 (29.3%) | 85 (92.4%) | NR | 24(17-38) | NR | NR | NR | NR | NR | NR |
| 7 | Danielsson, 2024 | Finland | Retrospective | 2009 – 2019 | AIH, PBC, PSC | ICD | 585 | US | 101 (15.6%) | 310 (47.8%) | NR | 26.6 | 27 (15.3) | 63 (35.8) | 70 (39.8) | NR | NR | NR |
| 8 | Davis, 2019 | USA | Prospective | NR | AIH | Biopsy | 61 | Biopsy | 19 (31.1%) | NR | NR | 28.3 (7) | NR | NR | NR | NR | NR | NR |
| 9 | De Luca-Johnson, 2016 | USA | Retrospective | 1973 – 2011 | AIH | Biopsy | 73 | Biopsy | 22 (30.1%) | 45 (61.6%) | NR | NR | 12 (16.4) | 33 (45.2) | 20 (27.4) | AIH-HS: 4 (80); AIH: 20 (71.4) | NR | NR |
| 10 | Del Barrio Azaceta, 2024 | Spain | Retrospective | NR | PBC | ICD | 436 | VCTE/US | 126 (28.9%) | 391 (89.1%) | NR | 25.8 | 94 (21.6) | 148 (33.9) | 129 (29.6) | NR | NR | NR |
| 11 | Dominguez Cardoso, 2022 | Mexico | Retrospective | 2015 – 2022 | PBC | Clinical | 60 | VCTE | 18 (20.0%) | 57 (90%) | NR | NR | NR | NR | NR | NR | NR | NR |
| 12 | Doycheva, 2014 | USA | Retrospective | 2000 – 2012 | PSC | Clinical | 81 | MRI | 7 (8.6%) | 22 (27.1%) | 57 (70.4%) | 25.6 (5) | 9 (11.1) | NR | NR | NR | NR | NR |
| 13 | Flatley, 2023 | UK | Retrospective | NR | AIH | Clinical | 354 | Biopsy | 89 (25.1%) | 287 (81.1%) | NR | 27.8 | 32 (9.0) | NR | NR | NR | NR | NR |
| 14 | Gatselis, 2021 | Greece | Retrospective | NR | AIH | Clinical | 186 | Biopsy | 49 (24.5%) | 142 (71.0%) | 44 (22.0%) | NR | NR | NR | NR | NR | NR | NR |
| 15 | Hernández-Pérez, 2024 | Spain | Retrospective | Until mid-2017 | PBC | Biopsy | 129 | Biopsy | 36 (27.9%) | 114 (88.4*) | 1 (0.8%) | NR | 16 (12.4) | 30 (23.3) | 52 (40.3) | PBC-HS: 17 (47.2); PBC: 70 (75.3) | NR | NR |
| 16 | Hindi, 2013 | USA | Retrospective | NR | PBC | ICD | 49 | Biopsy | 28 (57.1%) | 48 (98.0%) | NR | 25 (18-36) | 3 (6.1) | 7 (14.3) | 27 (55.1) | NR | NR | NR |
| 17 | Kaviani, 2024 | Canada | Retrospective | 1984 -2023 | PBC | Clinical | 115 | VCTE | 38 (33.0%) | 100 (87.0%) | 20 (17.4%) | NR | NR | NR | NR | NR | NR | PBC-HS: 3 (7.9); PBC: 4 (5.2) |
| 18 | Kilani, 2025 | USA | Retrospective | NR | AIH | ICD | 4798 | ICD | 1480 (30.8%) | 3512 (73.2%) | NR | NR | NR | NR | NR | NR | AIH-HS: 30 (2.1);  AIH: 15 (1.0) | NR |
| 19 | Lasyte, 2025 | Sweden | Prospective | NR | PSC | Clinical | 220 | VCTE | 64 (29.1%) | NR | NR | NR | NR | NR | NR | NR | NR | NR |
| 20 | Lim, 2025 | Korea | Retrospective | 2015 -2020 | AIH | ICD | 3805 | US | 826 (21.7%) | 108 (24.6%) | NR | NR | NR | NR | NR | NR | AIH-HS: 20 (2.4); AIH: 41 (1.4) | AIH-HS: 63 (7.6); AIH: 147 (4.9) |
| 21 | Liu K, 2024 | China | Retrospective | 2011 – 2021 | AIH | Clinical | 283 | Biopsy | 56 (25.2%) | 196 (88.3%) | 53 (23.9%) | 23.0 (3.3) | 67 (23.7) | 144 (50.9) | 146 (51.6) | NR | NR | NR |
| 22 | Liu P, 2022 | China | Retrospective | 2016 – 2022 | AIH | Biopsy | 222 | Biopsy | 65 (23.0%) | 233 (82.3%) | NR | 23.01 (3.16) | 31 (14.0) | 44 (19.8) | NR | AIH-HS: 31 (88.6); AIH: 33 (86.8) | NR | NR |
| 23 | Lytvyak, 2023 | Canada | Retrospective | 1971 - 2022 | AIH | Clinical | 228 | VCTE | 52 (22.8%) | 164 (71.9%) | 39 (17.1%) | NR | NR | NR | NR | NR | NR | NR |
| 24 | Lytvyak, 2025 | International | Retrospective | NR | PBC | Clinical | 820 | VCTE | 186 (22.7%) | 729 (88.9%) | NR | 27.0 | NR | NR | NR | PBC-HS: 147 (79.0)  PBC: 441 (70.0) | NR | NR |
| 25 | Mantaka, 2018 | Greece | Retrospective | NR | PBC | Clinical | 171 | Clinical | 46 (26.9%) | 148 (86.5%) | NR | 27.2 (4.85) | 33 (19.3) | NR | NR | NR | NR | NR |
| 26 | Marenco-Flores, 2023 | USA | Prospective | NR | PSC | ICD | 156 | Biopsy/US | 71 (45.5%) | 74 (47.4%) | NR | NR | NR | NR | NR | NR | NR | NR |
| 27 | Mederacke, 2020 | Germany | Retrospective | 2000 - 2014 | AIH | Clinical | 215 | Biopsy | 34 (14.2%) | 177 (74.1%) | 64 (26.8%) | 24.8 | 24 (10.0) | NR | NR | NR | NR | NR |
| 28 | Minuk, 2017 | Canada | Retrospective | NR | PBC | Clinical | 168 | US | 68 (28.6%) | 214 (90.7%) | NR | NR | NR | NR | NR | NR | NR | NR |
| 29 | Ni, 2021 | China | Retrospective | 2016 -2018 | AIH | Clinical | 114 | Clinical | 61 (53.5%) | 48 (78.7%) | NR | 23.97 (2.67) | NR | NR | NR | NR | NR | NR |
| 30 | Olivas, 2025 | Spain | Retrospective | NR | AIH | Clinical | 293 | VCTE | 91 (31.1%) | 206 (70.3%) | NR | NR | 33 (11.3) | NR | 64 (12.8) | NR | NR | NR |
| 31 | Ranginani, 2022 | USA | Retrospective | NR | PBC | ICD | 318 | VCTE | 62 (19.5%) | 288 (90.6%) | NR | 29.5 (6.7) | 57 (17.9) | 160 (50.3) | NR | NR | NR | NR |
| 32 | Ren, 2025 | China | Retrospective | 2018-2023 | PBC | Clinical | 363 | Imaging | 87 (24.0%) | 316 (87.1%) | 199 (54.8%) | NR | 29 (8.0) | 58 (16.0) | NR | NR | NR | NR |
| 33 | Salmon, 2010 | UK | Retrospective | NR | AIH | Clinical | 99 | Biopsy | 24 (24.2%) | 82 (82.9%) | NR | NR | NR | NR | NR | NR | NR | NR |
| 34 | Scheetz, 2024 | USA | Retrospective | NR | AIH | Clinical | 538 | Biopsy/VCTE | 136 (25.3%) | 426 (79.2%) | 138 (25.7%) | 30.1 | 64 (11.9) | 127 (23.6) | 53 (9.9) | NR | NR | NR |
| 35 | Sierra, 2023 | USA | Prospective | NR | PBC | Clinical | 216 | Biopsy/Imaging | 87 (40.3%) | NR | NR | NR | NR | NR | NR | PBC-HS: 74 (85.1); PBC: 107 (82.9) | NR | NR |
| 36 | Sorrentino, 2010 | Italy | Retrospective | NR | PBC | Clinical | 274 | Biopsy/Imaging | 111 (40.5%) | NR | NR | NR | NR | NR | NR | NR | NR | NR |
| 37 | Steinmann, 2024 | Germany | Retrospective | 2015 – 2020 | AIH, PBC, PSC | Biopsy | 354 | Biopsy | 90 (20.8%) | 304 (70.2%) | 112 (25.9%) | NR | 41 (9.5) | NR | NR | NR | NR | NR |
| 38 | Strzepka, 2024 | USA | Retrospective | 2009 -2015 | AIH | ICD | 51 | ICD | 26 (51.0%) | 41 (80.4%) | NR | NR | 10 (19.6) | 26 (51.0) | 18 (35.3) | NR | NR | NR |
| 39 | Takahashi, 2018 | Japan | Retrospective | 2009 – 2013 | AIH | Clinical | 1151 | Biopsy | 196 (17.0%) | 1007 (87.5%) | 73 (6.3%) | NR | NR | NR | NR | NR | AIH-HS: 3 (1.5); AIH: 10 (1.1) | NR |
| 40 | William, 2023 | USA | Retrospective | NR | PBC | ICD | 319 | VCTE | 52 (32.5%) | NR | NR | 33.2 | NR | NR | NR | NR | NR | NR |
| 41 | Yuan, 2024 | China | Retrospective | 2019 – 2022 | PBC | Clinical | 108 | Clinical | 117 (62.6%) | 133 (71.1%) | NR | NR | 23 (12.3) | 73 (39.0) | NR | NR | NR | NR |
| 42 | Zachariah, 2025 | UK | Retrospective | NR | PBC | Clinical | 115 | VCTE/Imaging/Biopsy | 38 (33.0%) | 103 (89.6%) | NR | NR | NR | NR | NR | NR | NR | NR |
| 43 | Zachou, 2022 | Greece, Canada, Japan, Germany, Netherlands, Spain | Retrospective | 2017 – 2019 | AIH | Clinical | 640 | Biopsy | 146 (22.8%) | 474 (74.1%) | 132 (20.6%) | NR | 124 (19.4) | 175 (27.3) | 264 (41.3) | NR | AIH-HS: 3 (2.6); AIH: 11 (2.1) | AIH-HS: 21 (18.3); AIH: 55 (10.5) |
| 44 | Zhao, 2023 | China | Retrospective | 2008 -2019 | PBC | Clinical | 789 | ICD | 17 (2.2%) | 689 (87.3%) | 454 (57.5%) | NR | 94 (11.9) | 126 (16.0) | 271 (34.3) | NR | PBC-HS: 1 (5.9); PBC: 16 (2.1) | PBC-HS: 1 (5.9); PBC: 82 (10.6) |

*Values reported as mean (standard deviation) or median (range) where appropriate

BMI: body mass index; HCC hepatocellular carcinoma; NR: not recorded; VCTE: vibration-controlled transient elastography

**Figure S1: PRISMA flowchart**

**
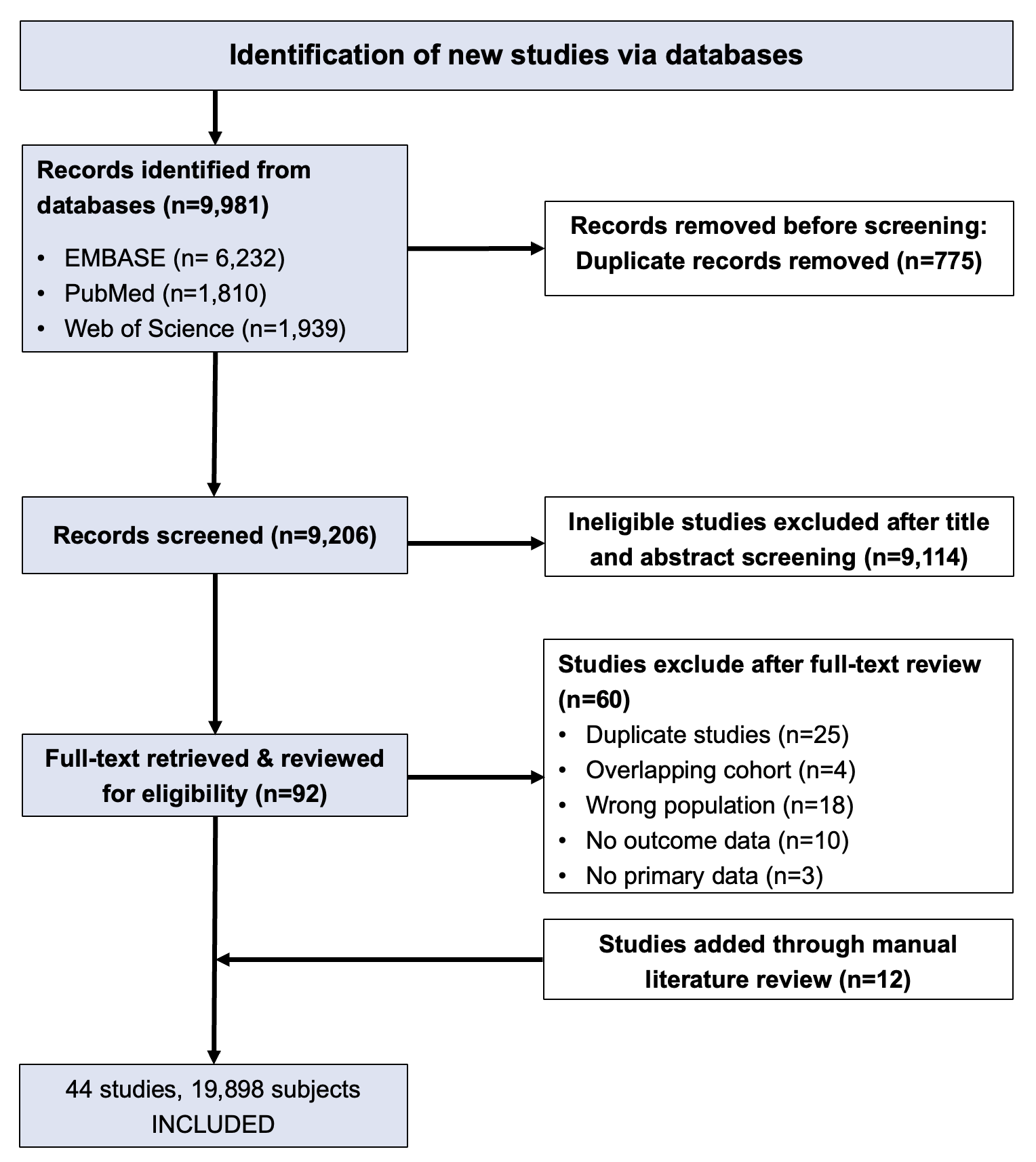
**

**Figure S2:** Traffic light plot summarizing risk-of-bias of all included studies


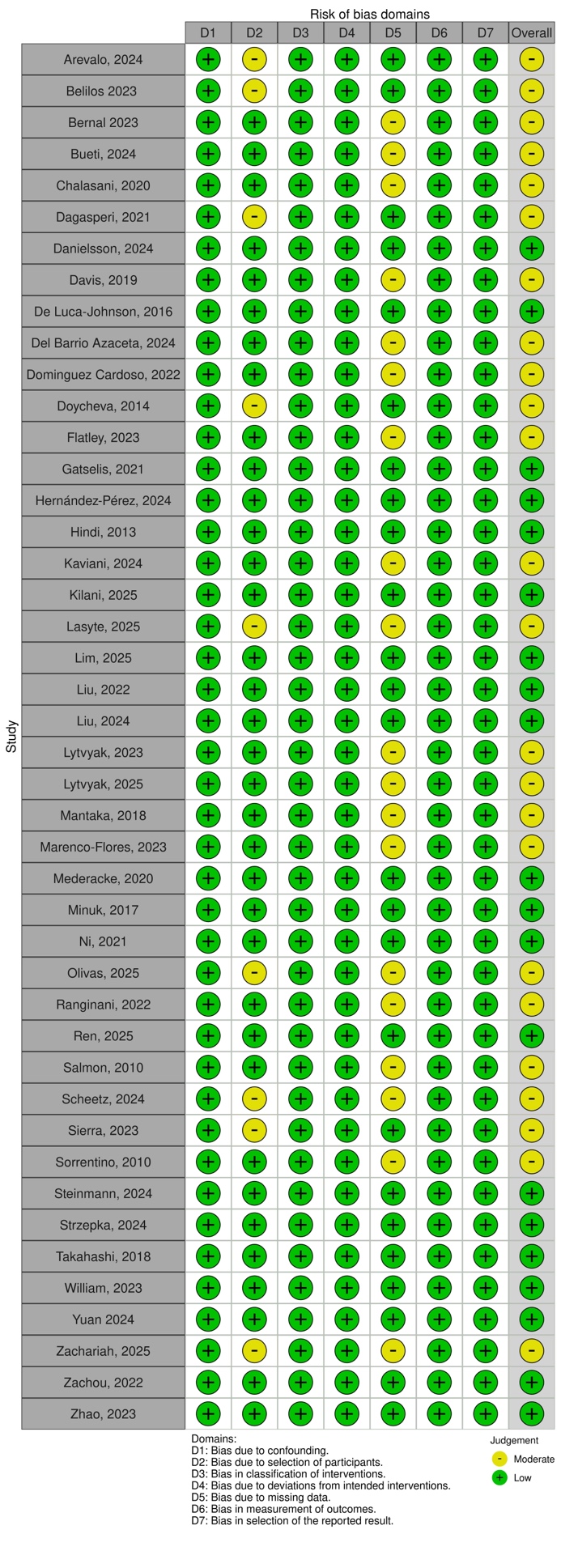


**Figure S3**: Forest plot showing effects of hepatic steatosis in AIH and PBC patients on undergoing **liver transplantation**


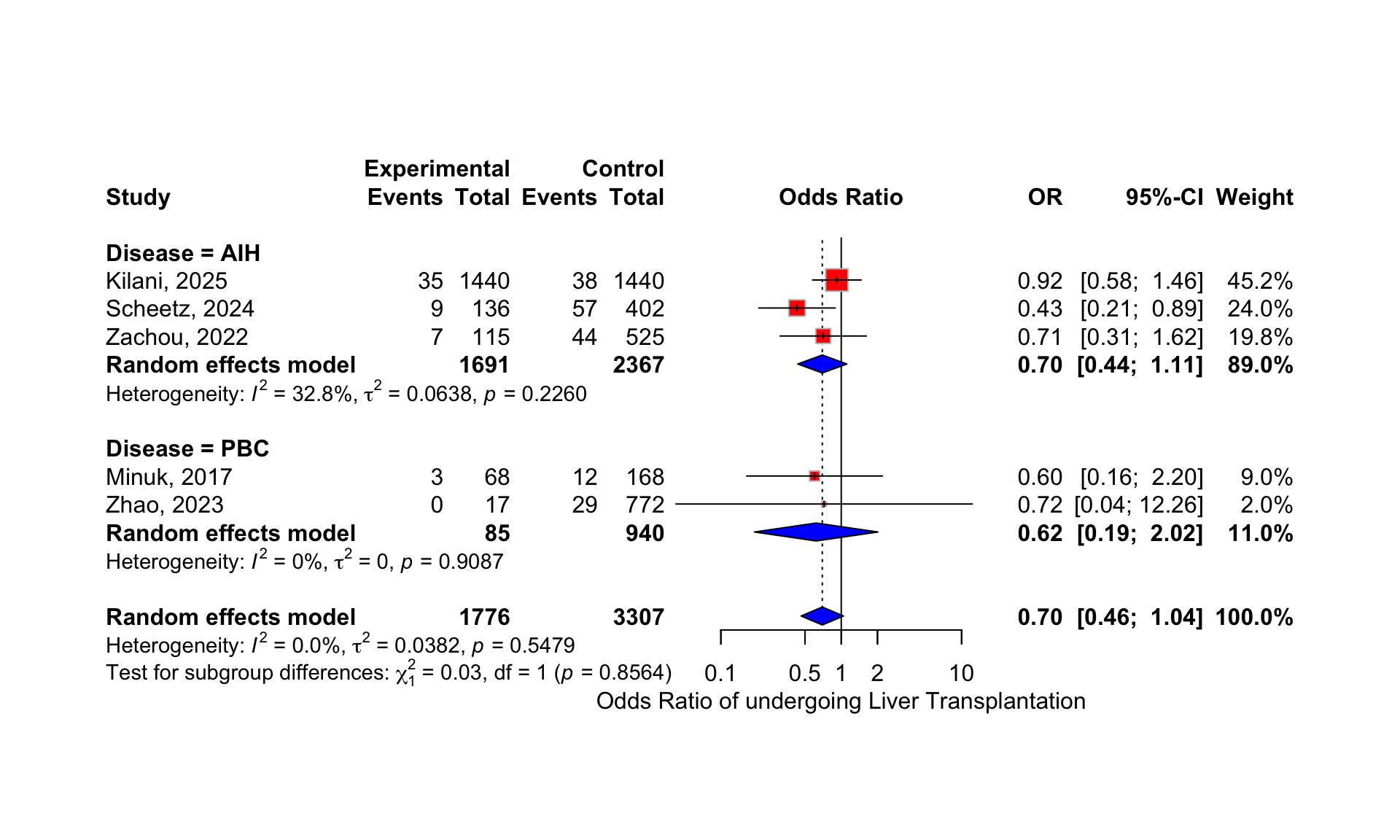


*HS: hepatic steatosis; AIH: autoimmune hepatitis; PBC: primary biliary cholangitis*

**Figure S4**: Forest plot showing the effects of hepatic steatosis in AIH and PBC patients on **all-cause mortality**.

**
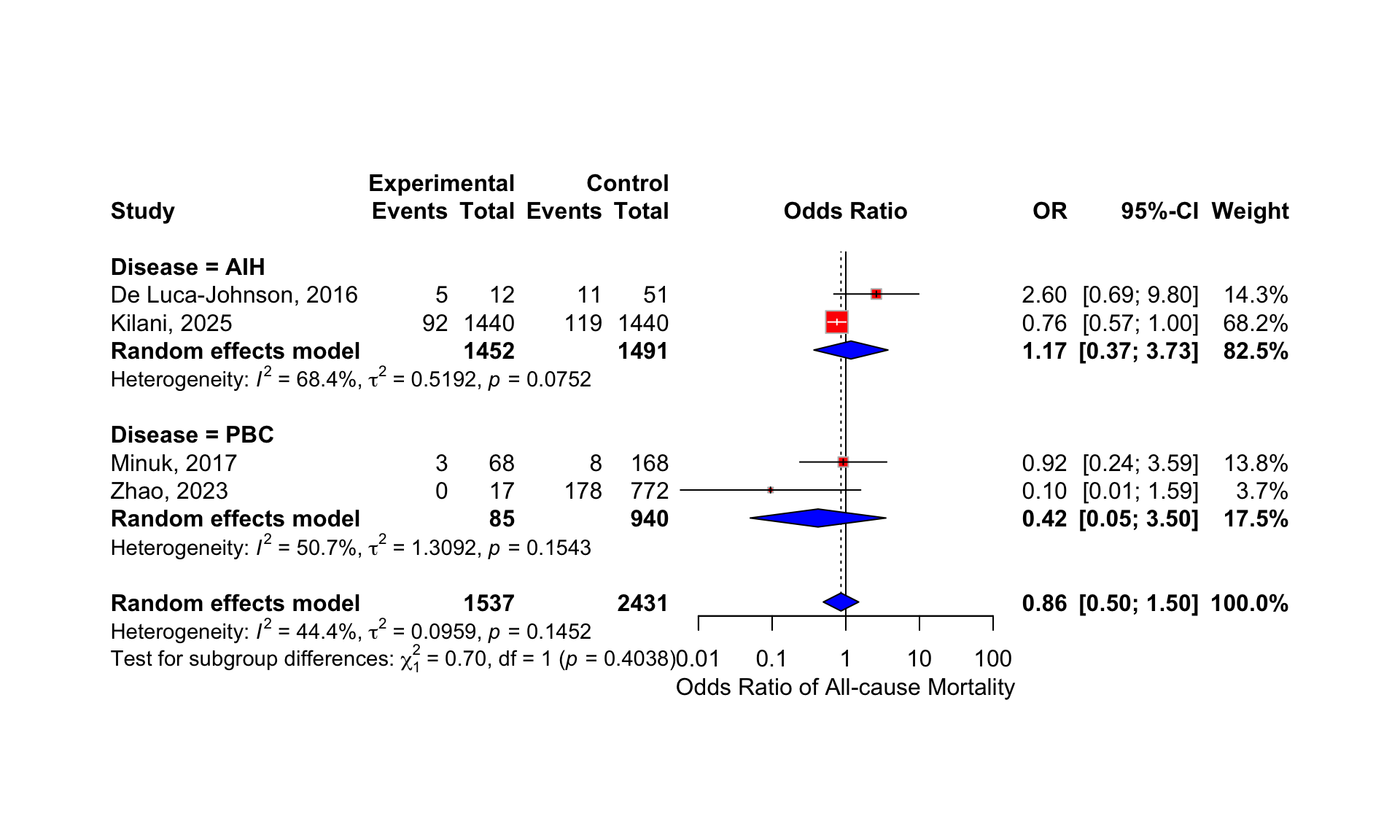
**

*HS: hepatic steatosis; AIH: autoimmune hepatitis; PBC: primary biliary cholangitis*

**Figure S5:** Forest plot showing the effects of hepatic steatosis in AIH and PBC patients on **treatment response**


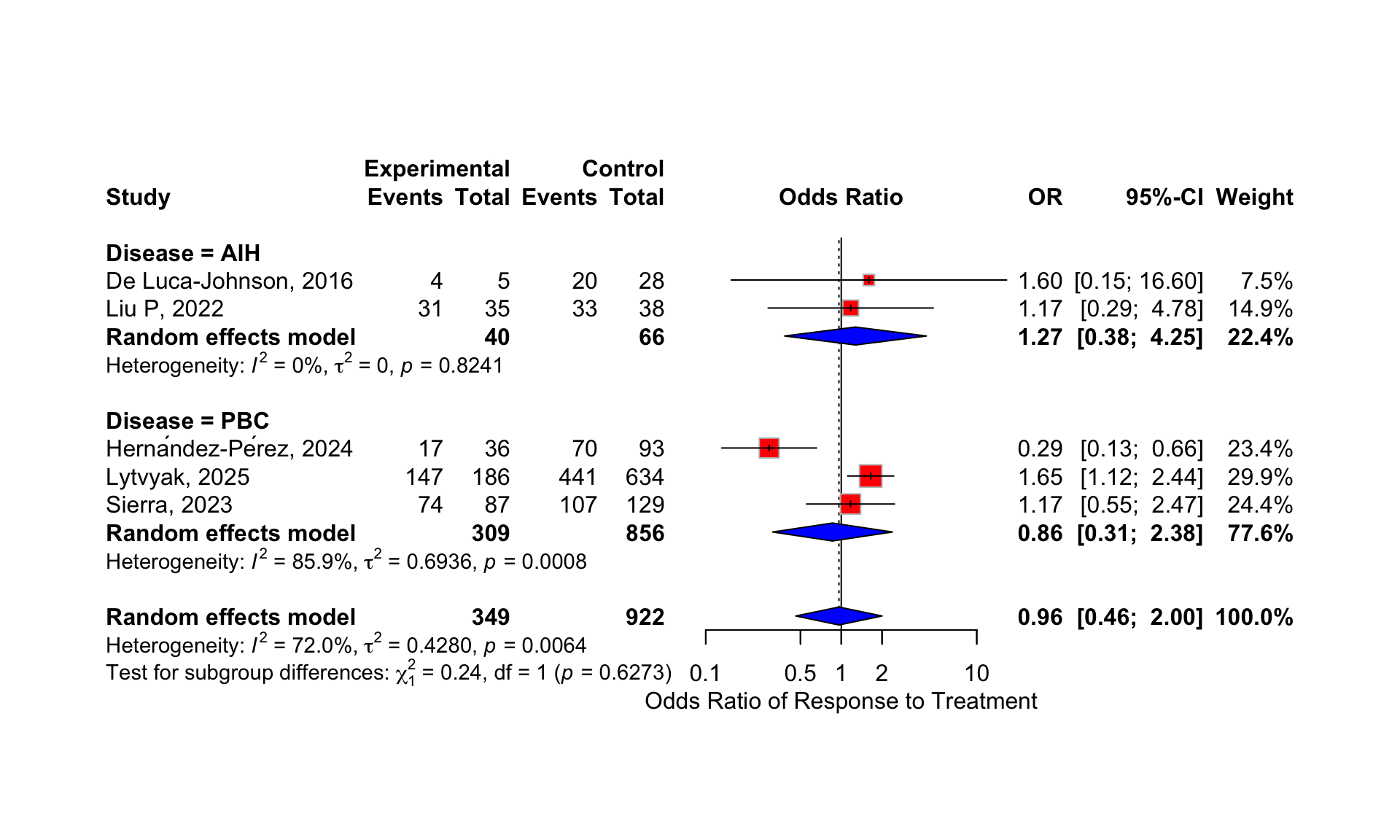


*HS: hepatic steatosis; AIH: autoimmune hepatitis; PBC: primary biliary cholangitis*

**Figure S6:** Leave-one-out analysis showing serial exclusion of individual studies did not alter the pooled estimates of hepatic steatosis in AILD

**
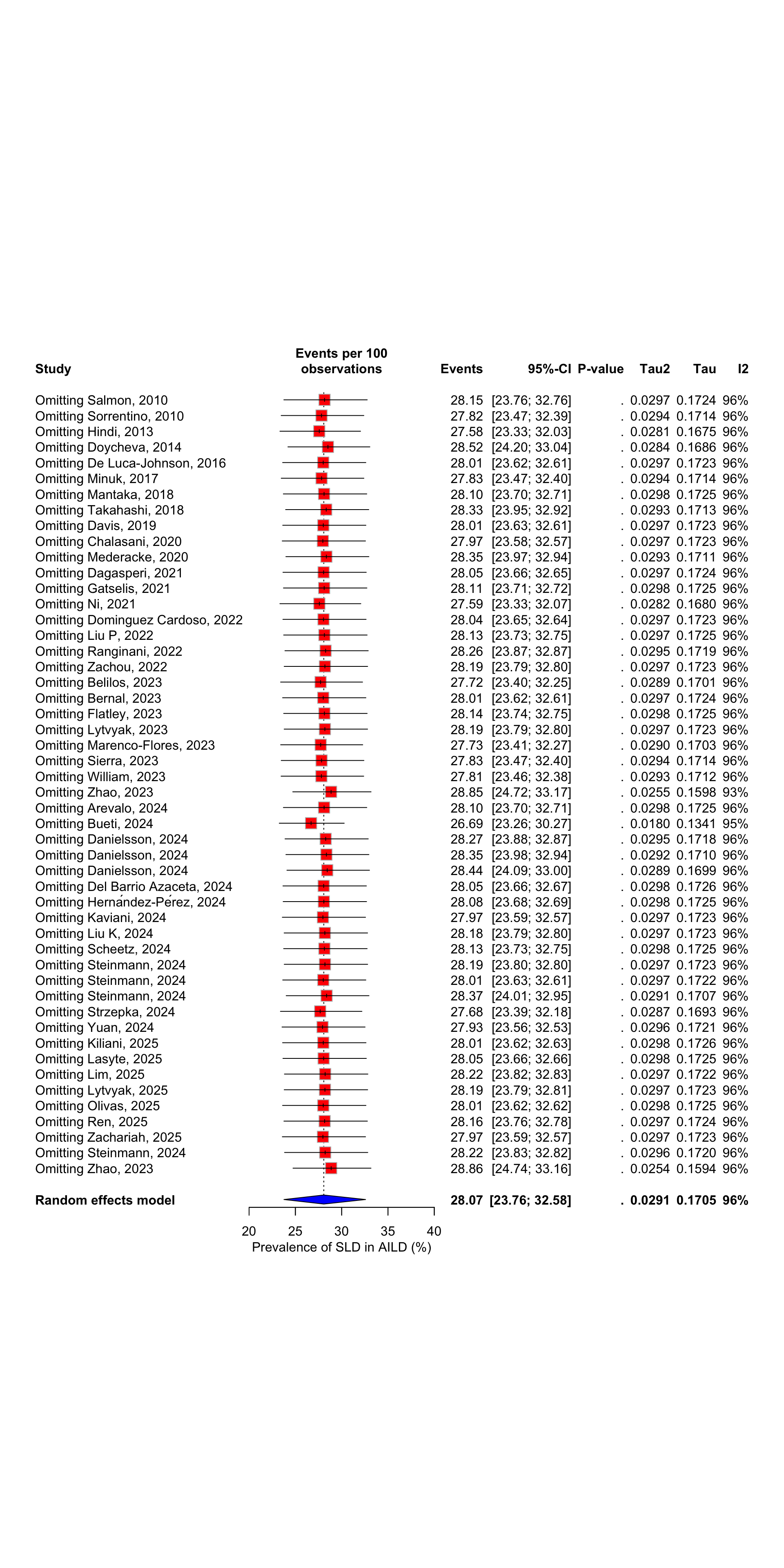
**

**Figure S7**: Sensitivity analysis of hepatic steatosis prevalence by method of hepatic steatosis diagnosis (biopsy versus non biopsy) in patients with A) Autoimmune hepatitis, and B) Primary biliary Cholangitis **
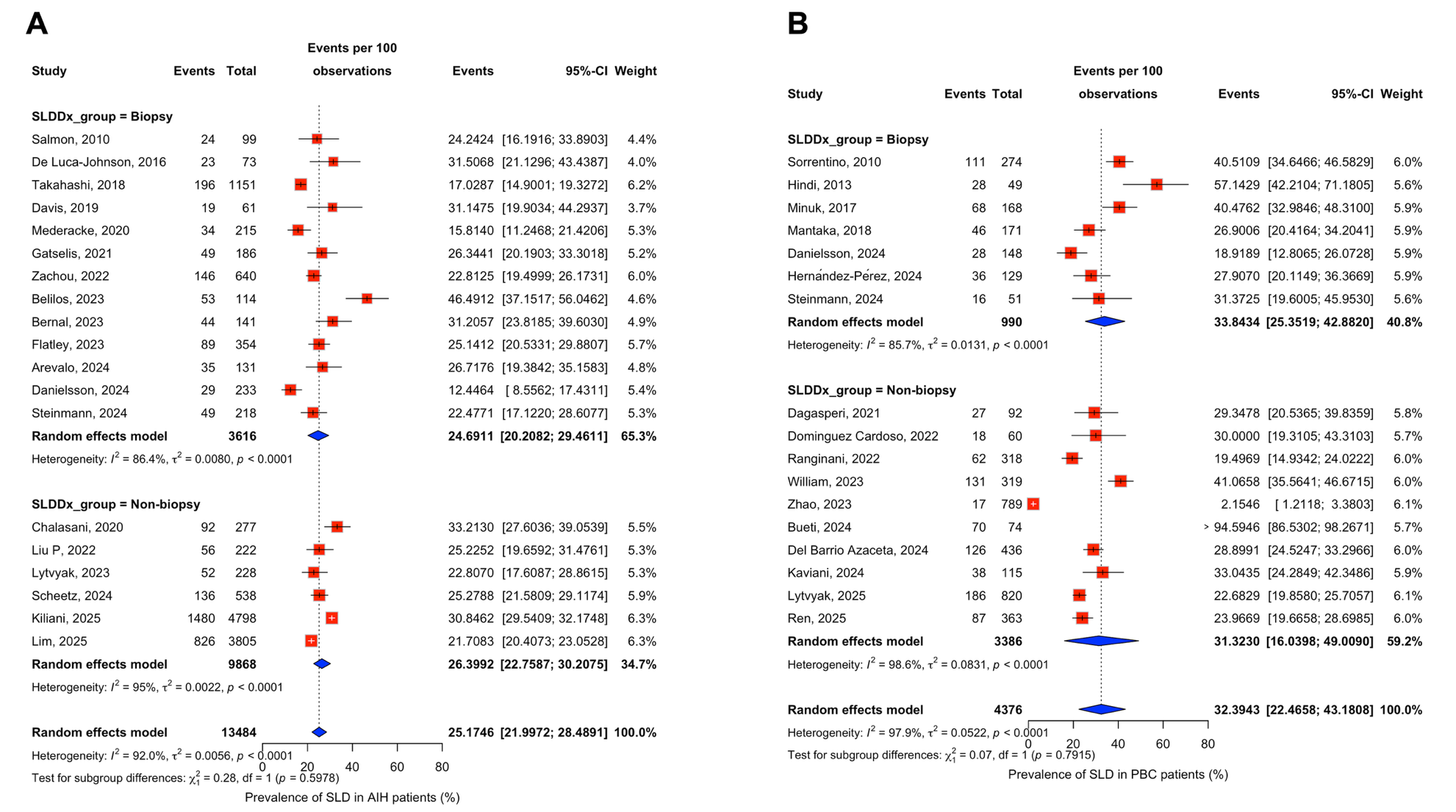
***HS: hepatic steatosis; AIH: autoimmune hepatitis; PBC: primary biliary cholangitis*

**Supplementary Figure S8**: Meta-regression of prevalence of hepatic steatosis in AIH and PBC against **A**: BMI (kg/m^2^); **B**: Type 2 Diabetes Mellitus; **C**: Hypertension; **D**: Dyslipidemia


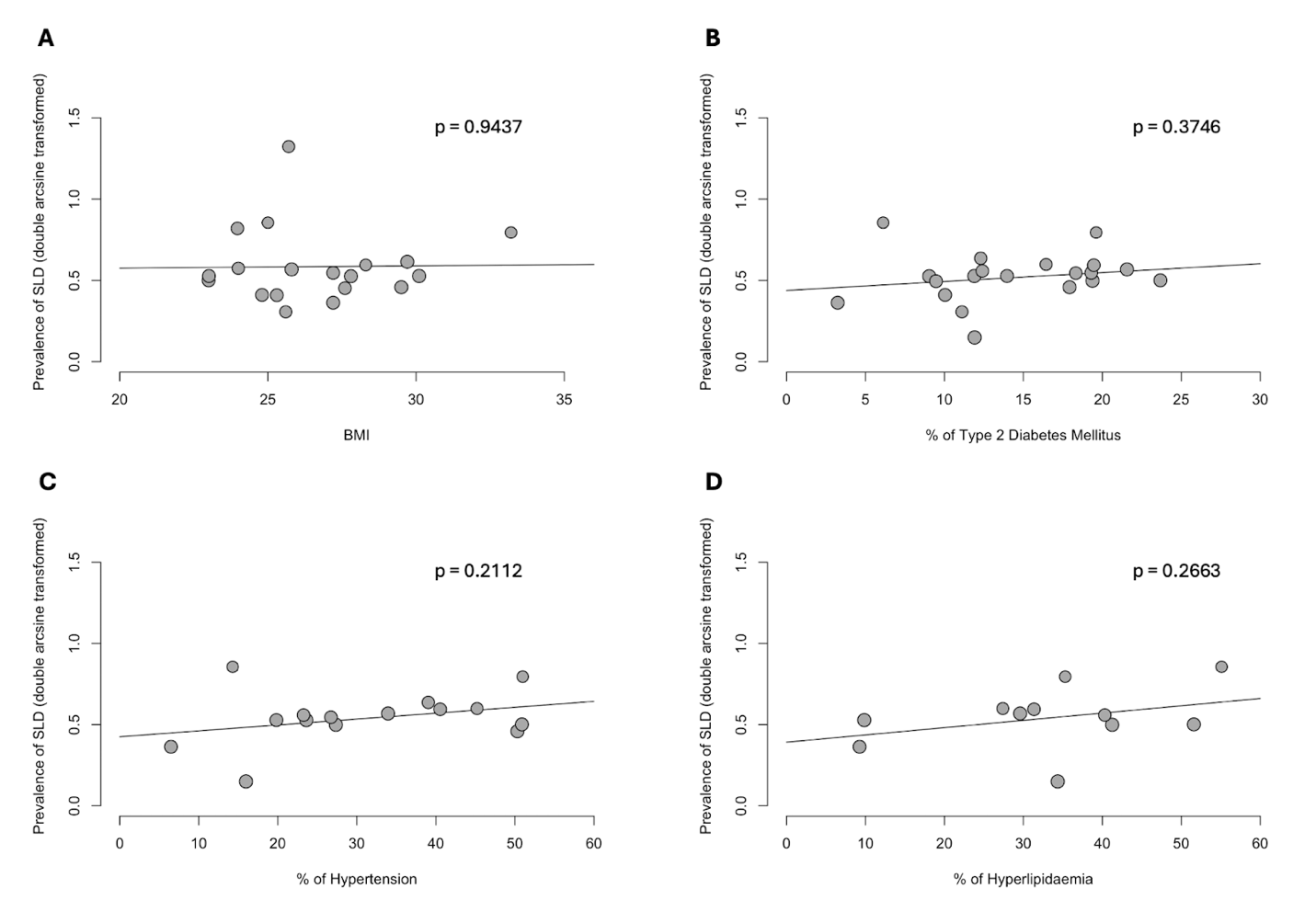


*HS: hepatic steatosis; AIH: autoimmune hepatitis; PBC: primary biliary cholangitis*

**Figure S9:** Funnel Plot on the pooled prevalence of HS in autoimmune hepatitis

**
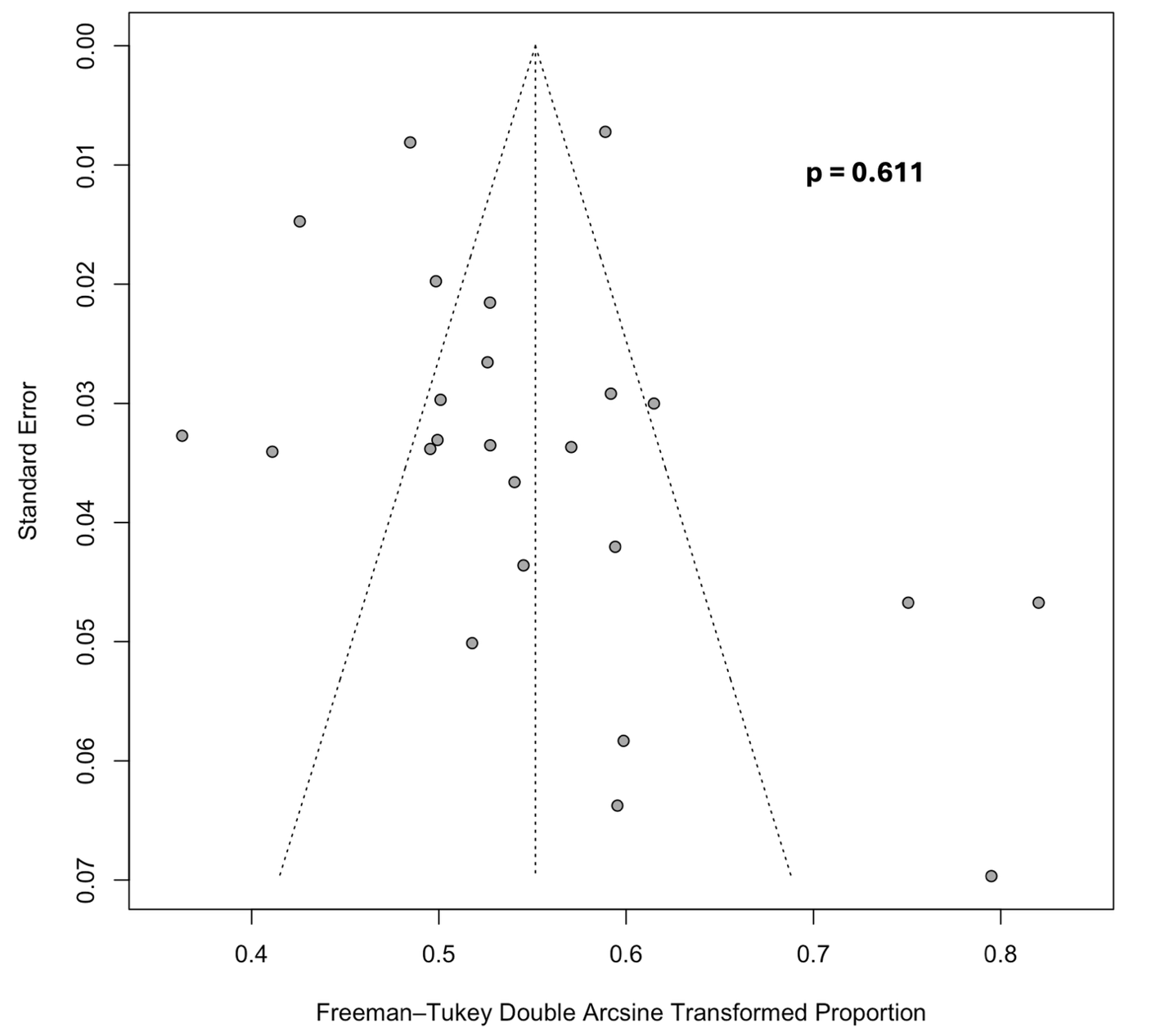
**

**Figure S10:** Funnel Plot on the pooled prevalence of HS in primary biliary cholangitis


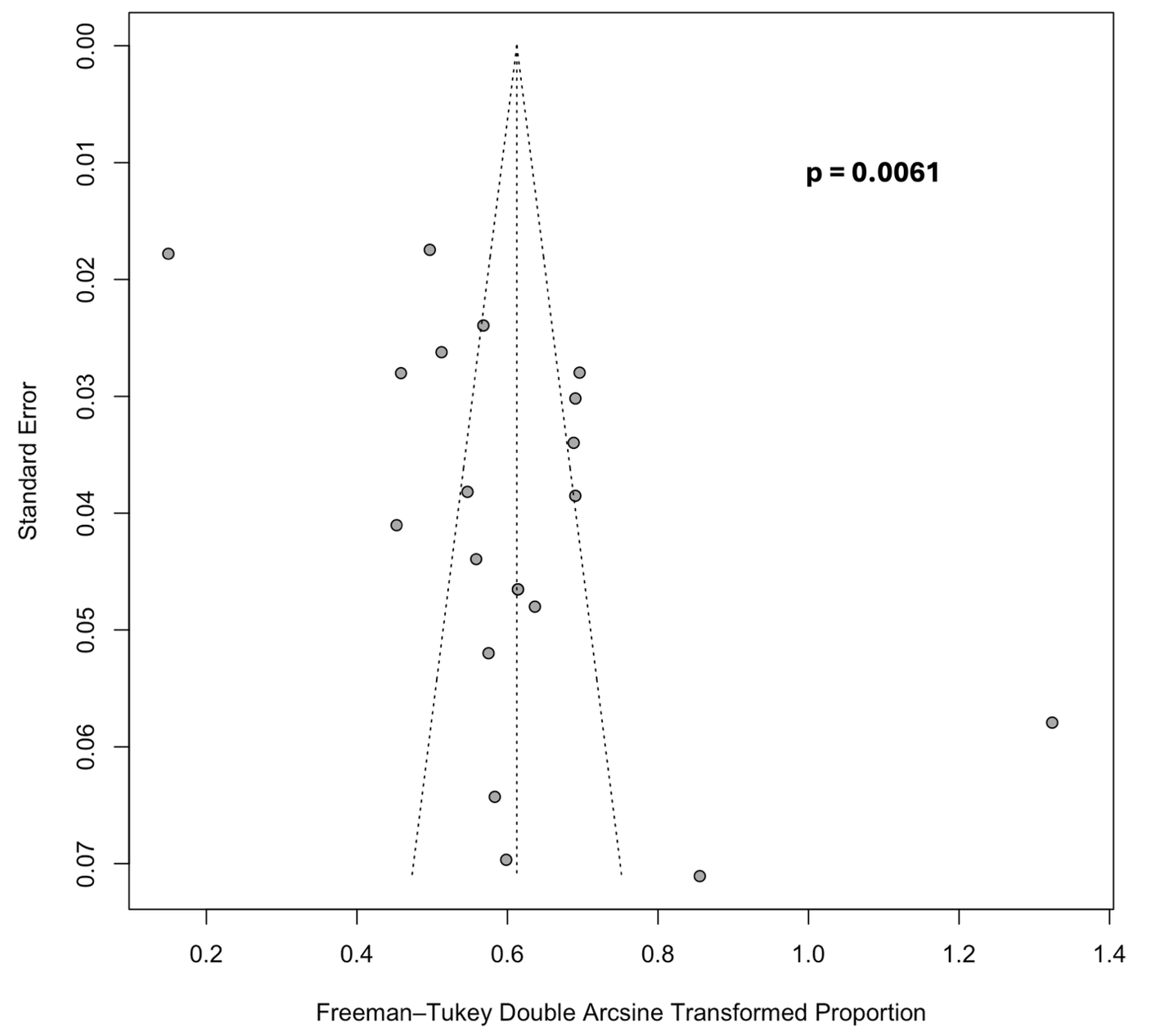

Supplement: Supplementary file 1 [file hc9-10-e0959-s001.docx]
